# Repurposing Drugs to Treat Heart and Brain Illness

**DOI:** 10.3390/ph14060573

**Published:** 2021-06-16

**Authors:** Maranda S. Cantrell, Alejandro Soto-Avellaneda, Jackson D. Wall, Aaron D. Ajeti, Brad E. Morrison, Lisa R. Warner, Owen M. McDougal

**Affiliations:** 1 Biomolecular Sciences Ph.D. Program, Boise State University, Boise, ID 83725, USA; marandacantrell@u.boisestate.edu (M.S.C.); AlexSoto@u.boisestate.edu (A.S.-A.); 2 Department of Chemistry and Biochemistry, Boise State University, Boise, ID 83725, USA; jacksonwall@u.boisestate.edu (J.D.W.); aaronajeti@u.boisestate.edu (A.D.A.); 3 Department of Biology, Boise State University, Boise, ID 83725, USA

**Keywords:** drug repurposing, drug development, cardiovascular disease, neurodegenerative disease, inflammation, drug repositioning

## Abstract

Drug development is a complicated, slow and expensive process with high failure rates. One strategy to mitigate these factors is to recycle existing drugs with viable safety profiles and have gained Food and Drug Administration approval following extensive clinical trials. Cardiovascular and neurodegenerative diseases are difficult to treat, and there exist few effective therapeutics, necessitating the development of new, more efficacious drugs. Recent scientific studies have led to a mechanistic understanding of heart and brain disease progression, which has led researchers to assess myriad drugs for their potential as pharmacological treatments for these ailments. The focus of this review is to survey strategies for the selection of drug repurposing candidates and provide representative case studies where drug repurposing strategies were used to discover therapeutics for cardiovascular and neurodegenerative diseases, with a focus on anti-inflammatory processes where new drug alternatives are needed.

## 1. Introduction

Drug development is a long, labor-intensive process with no guarantee of success. On average, it takes 10 years and 2.6 billion dollars to develop a new drug, with success rates averaging only about 12% [1]. One way to mitigate the barriers of drug development is to repurpose drugs approved by the Food and Drug Administration (FDA) as well as European Medicines Agency (EMA) for the treatment of different diseases. Drug repurposing is the practice of finding new uses for existing drugs at any stage of development. The benefit of drug repurposing is that the risk of failure is significantly lower than drug development, which permits more effective use of resources to optimize drug efficacy for treatment of the desired ailment. Much of the primary research regarding repurposed drug safety, mechanism, and dosage has already been completed when the drug was first studied [2]. For example, a drug that demonstrated efficacy in animal models with low side effects but failed in human clinical trials to perform its intended purpose, might be a good candidate for use as a therapeutic for a different disease. Drugs with the potential to be repurposed have progressed through many of the steps required to meet regulatory safety and potency benchmarks, allowing for an accelerated and abbreviated path to FDA approval. A few representative examples of successful drugs that have been repurposed include amantadine, acetylsalicylic acid, cyclosporine, minoxidil, and imatinib (Table 1) [3]. Repurposed drugs are generally screened by computer search algorithms from databases of therapeutics that may or may not have been sufficiently effective in providing treatment for the pathology that served as their original target. For example, some drugs lack sufficient drug-like qualities to warrant further investigation for treating an intended illness, but modification of the molecular scaffold or functional groups attached to the drug template may afford attributes and activity suitable for therapeutic viability. The therapeutic index for a repurposed drug requires investigation into dosage recommendations for maximal benefit to counter the illness, while minimizing detrimental side-effects to the patient.

### 1.1. Technology to Repurpose Drugs

Computational screening of previously marketed drugs to identify drug repurposing candidates can lead researchers to initiate new clinical trials for the treatment of alternative ailments. Multiple computational strategies have emerged to identify potential candidates for drug repurposing. Of these, four strategies have gained popularity in recent years and will be discussed in brief. These strategies are side effect similarity mapping, genome-wide association studies (GWAS), small-molecule peptide-influenced drug repurposing (SPIDR), and computational high-throughput screening (HTS) [9,10,11].

In side-effect similarity mapping, existing drugs are categorized by their side effects using the Unified Medical Language System (UMLS) ontology for medical symptoms. Developed by Campillos et al. (2008), 746 marketed drugs were organized according to their listed phenotypic side effect similarities rather than a chemical similarity, and 20 of these marketed drugs were confirmed through in vitro assays to indicate that side effect similarity may be indicative of common protein targets. Using this strategy, prediction of the likelihood that two drugs had the same target based on their side effects was demonstrated [12]. This study describes a process by which drugs marketed for different targets could be identified as having potential for repurposing.

GWAS identifies single nucleotide polymorphisms (SNPs) and their associated phenotypes for individuals with a given disease. GWAS is performed by genotyping individuals who share a common disease and determining whether a genetic variant is shared to statistical significance among this population. Experimental validation by chromatin precipitation is then performed and variants are associated with phenotypic effects [13]. This technique has been used to identify many novel variants to trait associations in a variety of diseases such as macular degeneration, anorexia nervosa, depression, coronary artery disease and diabetes mellitus, and can be used to determine novel drug targets as well [13,14]. For example, GWAS have been used to identify SNPs for Parkinson’s disease (PD) and determined that estradiol may protect dopaminergic neurons in PD. This supported previous evidence that estradiol may be protective against PD because men are more likely to be diagnosed [15]. The neuroprotective role of estradiol was later confirmed in in vivo studies. At present, estradiol is still not an approved treatment for PD, but efficacy studies are ongoing [2,16,17]. Another example of GWAS being useful for determining novel drug targets was the identification of a gene encoding a disintegrin and metalloproteinase with thrombospondin motifs TS7 (ADAMTS7), a gene implicated in coronary artery disease (CAD) for its presence in angiographic CAD patients and role in smooth muscle cell migration, a finding that supports the idea of genetically inherited CAD [16,18]. Using GWAS, it is possible to identify new potential targets based on SNPs.

SPIDR is a recently developed computational screening technique to identify drugs that can target a specific receptor by looking at its peptide ligand conformational space [11]. This method was integrated into the free program DockoMatic v 2.1, making this process accessible to lower-resourced institutions [17]. This technique works by first utilizing the genetic algorithm managed peptide mutant screening (GAMPMS) to search for peptide mutants with high binding affinity to the receptor, and then this population of peptides is moved over to the SimSearcher utility to identify the best small molecules analogous to the peptide population for binding to the isoform [11]. SPIDR was developed and used to find 12 small molecules computationally predicted to bind with high affinity to a nicotinic acetylcholine receptor (nAChR) isoform associated with Alzheimer’s and PD, by looking at α-conotoxin MII peptide analogs that would bind favorable to the receptor isoform specifically [11]. Following this publication, a qualitative assay to detect dopamine release by ligand action on nAChRs was developed with the intent to assess bioactivity of molecules that may act on different nAChR isoforms for drug development. This assay was created using a luminescence-based assay and validated with known nAChR binders: acetylcholine, nicotine and cytosine [19]. The PC-12 cell-based assay was intended to be used to test the small molecules found in the SPIDR development publication for nAChR activity as an in vitro validation for the SPIDR methodology.

Additionally, small molecule compound screens can be used to identify drugs amenable to being repurposed. Computational programs such as AutoDock Vina are often used to screen molecular library databases like ZINC, chEMBL, PubChem, DrugBank, and others [20,21,22]. Computational screens of small molecule databases are an ideal starting point for any drug discovery project to narrow down a list of compounds that may bind a biological target with high affinity. However, wet lab experimentation needs to be conducted, which can be done with commercially available libraries of drugs using in vitro cell-based assays. Researchers study whether a drug induces a phenotypic effect in cell-based assays, which are usually performed in cell culture or on a model organism whereas a target-based screen can be performed in vitro using techniques like enzyme-linked immunosorbent assay (ELISA), Surface Plasmon Resonance (SPR) or Nuclear Magnetic Resonance (NMR) spectroscopy. Many reviews have been recently published on the topic of drug repurposing in either CVD or neurodegeneration [23,24,25,26,27,28,29]. However, there is little explored in the realm of drugs that may be repurposed to target inflammatory processes which link these two diseases. In this article, we will briefly discuss strategies for repurposing drugs and examples of drugs that are being repurposed for treatment of cardiovascular disease and neurodegenerative disorders. Specifically, we will cover those conditions that affect both cardiovascular and neurodegenerative disease such as inflammatory pathways, which are intimately connected in heart and brain disease.

### 1.2. Heart and Brain Interconnectedness in Disease

There are currently few therapeutics for cardiovascular and neurodegenerative diseases that provide suitable efficacy and minimal side effects, creating ideal conditions for drug repurposing. Not only are cardiovascular and neurodegenerative diseases difficult to treat, but treatment options remain limited, and many drugs do not cease progression nor achieve remission of the disease state. Considerable progress has been made in recent years to understand the molecular determinants of cardiovascular disease, which encompasses a wide variety of disorders such as endothelial dysfunction, atherosclerosis and pulmonary arterial hypertension, and neurodegenerative disorders, including Parkinson’s and Alzheimer’s diseases [30,31] Cardiovascular disease and neurodegenerative disorders share a common trait of inflammation as a central component to the pathogenic process. Targeting inflammatory processes with repurposed drugs can have a multitude of beneficial effects for patients with diseases of both the heart and brain. Currently, repurposed drugs are being used in studies associated with two cardiovascular related diseases that include endothelial dysfunction and pulmonary arterial hypertension. In neurodegenerative diseases, repurposed drugs are being studied for the treatment of autophagy and neuroinflammation (Table 2).

## 2. Targeting Endothelial Dysfunction in Cardiovascular Disease

Cardiovascular diseases including atherosclerosis, coronary artery disease, and arrhythmias that eventually lead to myocardial infarction and/or stroke. Ischemic events such as these are the leading cause of death in the world [35]. Pharmacological intervention of cardiovascular diseases is provided by statins, beta-blockers, and angiotensin-converting enzyme inhibitors. In more advanced cases, surgical intervention may be necessary. The medications for cardiovascular disease target high cholesterol and low-density lipoprotein levels, and hypertension. There is an emerging field of research to discover small molecule inhibitors for alternative pathways aimed at the disruption of heart disease progression [36]. There is a robust market for pharmaceutical companies to create drugs to treat disorders, but the potential for repurposing already FDA-approved drugs constitutes a desirable alternative to discovery since these medications have been deemed safe enough for patient use and thus lower the clinical trial barrier for novel therapeutic usage.

Endothelial dysfunction is especially important in cardiovascular disease because it is a leading contributor to cardiac events. The endothelium is characterized by the cells of the tissue lining the various organs of the body, including the arteries and lymphatic system. The endothelium plays a major role in blood flow and constriction by facilitating the synthesis and degradation of vasodilating factors, such as nitric oxide (NO), arachidonic acid metabolites and reactive oxygen species (ROS) [37]. Endothelial dysfunction occurs when the endothelium fails to function properly. Namely, that the endothelium dysfunctions in the formation of vasodilating factors. Endothelial dysfunction is a component of atherosclerosis, which is characterized by the hardening and narrowing of the arterial walls, and hyperlipidemia, or high lipid concentrations in the blood.

Three drugs have been investigated for their potential to treat diseases of endothelial dysfunction that will be discussed here include colchicine, traditionally used to treat gout; methotrexate, an immunosuppressant and chemotherapy drug; and Tocilizumab, an immunosuppressant used to treat severe rheumatoid arthritis.

### 2.1. Colchicine

Colchicine is a secondary metabolite from the plants *Gloriosa superba* and *Colchicum autumnale*, which is toxic when ingested [38]. The current FDA and EMA approved use for colchicine is to treat gout, an inflammatory form of arthritis common in people with high levels of uric acid in their blood. Due to its anti-inflammatory properties, colchicine is currently under investigation as a potential endothelial dysfunction drug. Endothelial dysfunction is a characteristic of cardiovascular disease that leads to an increase in macrophages, T lymphocytes, and growth factors that contribute to atherosclerotic lesion formation associated with atherosclerosis [39,40,41,42]. The same inflammation inhibition mechanism that permits colchicine to be effective against gout, may also be applicable to the treatment of patients with early stages of atherosclerosis [43,44]. 

**Figure 1:**
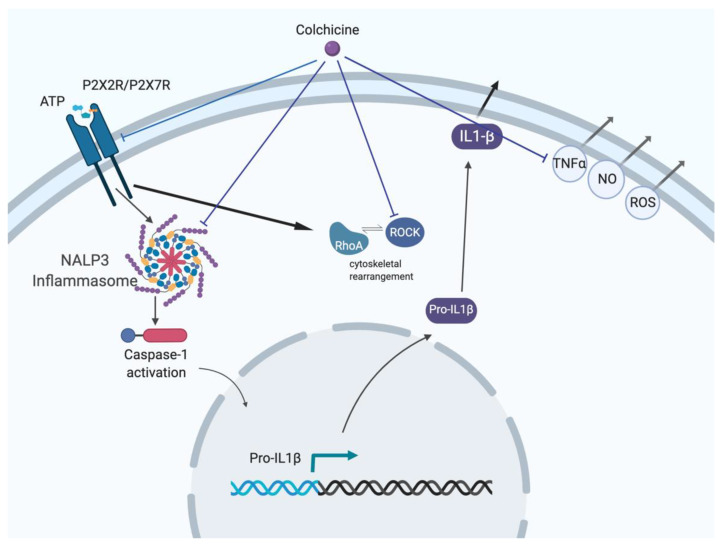
Inhibitory pathway of colchicine. Colchicine inhibits activation of purinergic P2X2/P2X7 receptors and blocks cation uptake and subsequent pro-inflammatory signaling cascades without affecting cell death. Colchicine also inhibits the NALP3 inflammasome pathway, the Rho/ROCK pathway via cytoskeleton rearrangement, and inhibits release of ROS, NO and TNFα. Figure 1 was created using BioRender.com (adapted from [45]).

Colchicine treats inflammation by binding to tubulin; preventing tubulin polymerization and function (see Figure 1) [17]. As a result, colchicine halts mitotic cells in metaphase. Colchicine also concentrates in neutrophils and prevents chemotaxis via the release of crystal-derived chemotactic factor, and inhibits monosodium urate induced loss of myeloid inhibitory C-type lectin-like receptor in neutrophils and interleukin-8 formation [45] Myeloid inhibitory C-type lectin-like receptor and interleukin-8 are important in immune homeostasis and inflammatory response [46,47].

Cell adhesion is the process by which cells connect to one another through the action of proteins, electrostatic interactions, and hydrophobic interactions. Adhesion is important because cells can use physical contact with one another to communicate via signal transduction. Colchicine interferes with cell adhesion by inducing the shedding of neutrophil adhesion molecules, which are important in neutrophil function [45]. These neutrophil adhesion molecules include selectins and counter on receptors that partake in signal transduction. Selectins are single-chain transmembrane glycoproteins organized by leukocyte (L), endothelial (E) and platelet/endothelial (P) selectins. They are characterized by their similar homology in both sequence, structure and sugar ligands. Colchicine reduces endothelial expression of selectins by acting on leukocytes in coronary artery disease [44]. 

Nidorf et al. reviewed ongoing clinical trial results and experimental evidence that supported the use of colchicine as an anti-inflammatory drug with promising efficacy toward the treatment of atherosclerosis [43]. First, they describe the mechanisms by which colchicine acts on the various inflammatory pathways including the production of pro-inflammatory cytokines, such as interleukin (IL)-1ß, reduction of platelet leukocyte interactions that lead to atherothrombosis and suppressing the growth of fibroblasts and osteophytes. At lower doses (0.5 mg/d), colchicine was found to have no reported side effects on patients with liver and/or kidney disease [43]. The authors concluded that colchicine could be repurposed for treatment of inflammation associated with atherosclerosis.

### 2.2. Methotrexate

**Figure 2:**
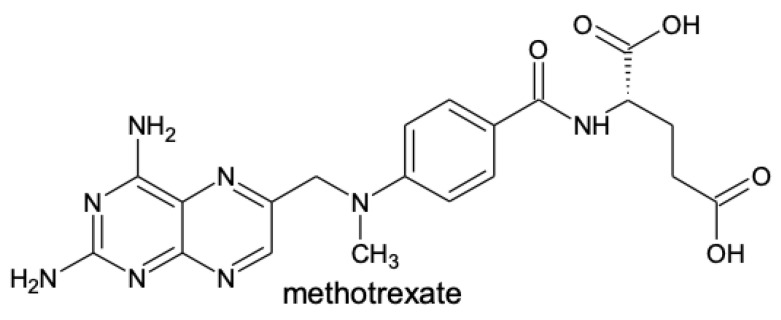
Chemical structure of methotrexate.

In the 1960s, the National Institutes of Health reported that methotrexate (Figure 2) was effective at treating rheumatoid arthritis and psoriatic arthritis. However, the rheumatology field at the time maintained a fidelity for corticosteroids to treat rheumatic syndromes, which stifled the use of methotrexate. Subsequent studies were conducted, establishing evidence that methotrexate was effective in the treatment of rheumatoid arthritis [48].

Methotrexate is a synthetic disease-modifying antirheumatic drug that is a structural analog of folic acid [31]. Methotrexate was originally used as a cancer drug but has recently been investigated for treatment of inflammation associated with endothelial dysfunction. In a study of 673 patients, methotrexate was used for the treatment of rheumatoid arthritis, and was determined that with proper dosing and use, it could be a beneficial drug to treat arthritis with 1.7% adverse side effect frequency and 0.15% mortality [49]. Methotrexate was identified as a good candidate for use to treat inflammatory diseases, including endothelial dysfunction. 

**Figure 3:**
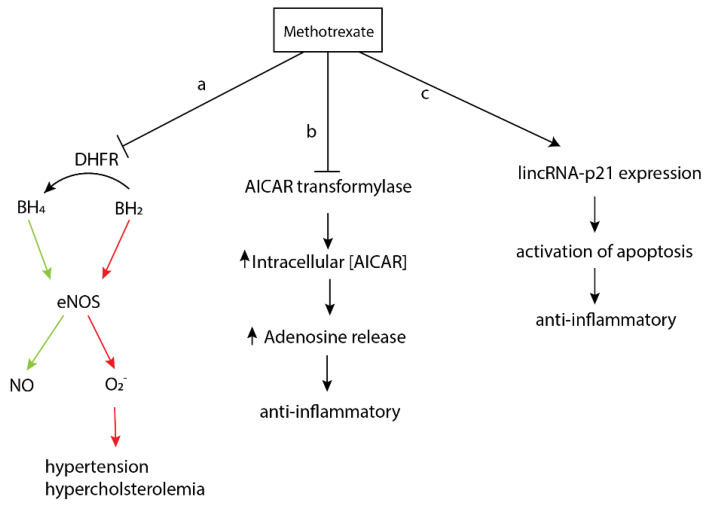
Metabolic pathways of methotrexate. (**a**) Methotrexate inhibits dihydrofolate reductase (DHFR) and prevents conversion of dihydrobiopterin (BH_2_) to tetrahydrobiopterin (BH_4_), leading to uncoupling of NO synthase. (**b**) Methotrexate inhibits AICAR transformylase, leading to increased adenosine levels and subsequent anti-inflammatory responses. (**c**) Methotrexate stimulates lincRNA-p21 expression, leading to increased apoptotic gene expression and subsequent anti-inflammatory responses.

Methotrexate exerts a multitude of biochemical and biological perturbations when administered to patients. While the exact or primary mechanism of action is not completely understood, multiple modes of activity have been characterized that reveal how methotrexate functions (Figure 3) [50]. First, methotrexate diminishes the immune response by increasing the rate of T-cell apoptosis. Methotrexate binds dihydrofolate reductase, causing an increase in nitric oxide synthase uncoupling through the prevention of the conversion of dihydrobiopterin to tetrahydrobiopterin (Figure 3a). Second, methotrexate suppresses inflammation and the immune response by promoting the release of adenosine. Metabolites of methotrexate inhibit aminoimidazole-4-carboxamide ribonucleotide (AICAR) transformylase, causing an increase in the concentration of intracellular AICAR and thus more adenosine release (Figure 3b). Third, methotrexate increases the expression of long intergenic non-coding RNA p21, a mediator and regulator of a variety of pro-apoptotic processes (Figure 3c) [51]. 

There is increasing evidence that the 5′-adenosine monophosphate-activated protein kinase (AMPK) is beneficial to the endothelium by protecting against apoptosis, oxidative stress and, increases NO production in the endothelium, which reduces cholesterol efflux activity [31]. Stimulation of AMPK as a treatment for endothelial dysfunction is under investigation. One study showed that the application of methotrexate on perivascular adipose tissue from Sprague-Dawley rats increased AMPK activity under basal and inflammatory conditions when treated with a negative control, palmitic acid [31]. A positive control using aminoimidazole-4-carboxamide ribonucleotide, a known AMPK agonist, was also able to stimulate AMPK activity [31]. This study similarly showed that methotrexate stimulated adiponectin mRNA expression and suppressed the pro-inflammatory molecules nuclear factor kappa B (NFκB) p-65, tumor necrosis factor alpha (TNF-α), and interleukin-6 (IL-6) activity [31]. At the time of this review, only studies of methotrexate on perivascular adipose tissue taken from sacrificed mice and in vitro studies in cell culture have been performed, necessitating investigation into more physiologically relevant in vivo model systems. 

On the contrary, other studies have indicated that methotrexate may contribute to vascular endothelial dysfunction by causing endothelial damage [52]. Methotrexate is a direct competitor with folic acid for the active site of human serum albumin, and indeed folic acid supplements are often provided in conjunction with methotrexate to prevent folic acid deficiency [53]. A study performed in Wistar rats, by Sankrityayan in 2016, showed that when administered with methotrexate, ex vivo vascular reactivity in the aorta was significantly reduced [52]. Vascular reactivity is the phenomenon responsible for both vasoconstriction and vasodilation, and those responses to various stimuli implicated in vasculature. Methotrexate has also been implicated in the reduction of NO, leading to increased oxidative stress and hindering of bioavailability of tetrahydrobiopterin by oxidizing it to dihydrobiopterin in the aorta [54,55]. This leads to hypertension, hyperlipidemia and induces endothelial dysfunction, because tetrahydrobiopterin is necessary as a cofactor required for the synthesis of NO [56].

There is contradicting evidence for methotrexate use as an endothelial dysfunction drug. On the one hand, there is data that suggests methotrexate reduces inflammation, while methotrexate also appears to lead to endothelial dysfunction. Therefore, more studies must be performed to determine the true mechanism of action for methotrexate on the vascular endothelium if it is to be used to treat endothelial dysfunction. There are currently 2140 clinical trials listed under the National Library of Medicine’s clinical trial database, with methotrexate being evaluated for the treatment of everything from ectopic pregnancy, to schizophrenia, to alopecia and cancer. It appears that in many pathologies where inflammation is a principal concern, or where immunomodulation is of utility, methotrexate may provide benefit. 

### 2.3. Tocilizumab 

Tocilizumab (TCZ) is an IL-6 inhibitor and recombinant monoclonal antibody used in rheumatoid arthritis treatment since obtaining EMA approval in 2009 and FDA approval in 2010 [57]. TCZ binds IL-6 and blocks JAK/STAT signaling to prevent the production of pro-inflammatory molecules [57,58]. It is currently being investigated for the treatment of endothelial dysfunction due to its anti-inflammatory properties. This is because RA is often listed as a co-morbidity in approximately 39–50% of atherosclerosis-related deaths, with endothelial dysfunction presented in many RA patients [59]. One hypothesis is that the release of networks of chromatin and granules (NETs) into the extracellular space, a common occurrence in both RA and endothelial function. TCZ was found to alleviate symptoms in endothelial dysfunction in a human clinical study [58].

## 3. Targeting Pulmonary Arterial Hypertension

Pulmonary arterial hypertension (PAH) is a relatively rare condition, affecting approximately 10–52 people per million population, characterized by the narrowing of the pulmonary arteries [60,61]. Left untreated, pulmonary arterial hypertension leads to the buildup of pressure on the right side of the heart when blood vessels in the lungs are diseased, causing the arteries to become increasingly narrow with time, and right-sided heart failure, often resulting in premature death [62,63]. While the direct cause of pulmonary arterial hypertension is unknown, a set of factors including liver disease, HIV infection, intravenous drug use, autoimmune disorders, and others are suspected to be culpable. For pulmonary arterial hypertension, there are several drugs under investigation for use that are in clinical trials [64]. Of these, two drugs—Anakinra and Ubenimex—target inflammatory processes specifically.

### 3.1. Anakinra

Anakinra was approved for use in rheumatoid arthritis by the FDA in 2001 and EMA in 2002 but is currently under investigation and has undergone Phase I clinical trial studies for novel use in PAH [64,65]. Anakinra, an interleukin-1 (IL-1) receptor protein antagonist analogue, is recombinantly expressed in *E. coli* and then administered via subcutaneous injection. The proposed mechanism of action for PAH, shown in mice, is to block perivascular macrophage recruitment in pulmonary artery smooth muscle cells [66]. In an inflammatory response pathway, IL-1b binds to IL-1 receptor (IL-1R) and recruits the myeloid differentiation primary response protein 88 (MyD88) and induces IL-1 synthesis via NFκB activation. In this study, the goal was to investigate whether this pathway played a role in the progression of PAH [66]. First, it was determined that in both the lung tissue taken from patients with PAH as well as mice with PAH, there were increased expression levels of both IL-1R and MyD88. Hypoxic wild-type mice treated with anakinra (20 mg/kg per day) were shown to have lower severity in PAH symptoms, namely, right ventricular systolic presser and hypertrophy, than untreated mice. These findings provided evidence that anakinra may be a good potential treatment for PAH. Furthermore, the pilot Phase I clinical study (clinicaltrials.gov: NCT03057028) done with treatment of PAH by anakinra found promising results.

### 3.2. Tadalafil

Tadalafil is currently used to treat erectile dysfunction and benign prostatic hyperplasia, and was approved by the EMA in 2008 and FDA in 2009 for treatment of pulmonary arterial hypertension [64,67]. Tadalafil is a phosphodiesterase type-5 (PDE5) inhibitor [68]. PDE5 mRNA levels are mostly found in the cerebellum, kidney, pancreas, and rat lung tissues, which may be indicative of human mRNA localization [69]. The result, here, is such that cyclic guanosine monophosphate (cGMP) concentrations are elevated and thus facilitate the NO pathway, namely vasodilation [70]. In brief, cGMP binds to PDE5 and increases catalytic activity approximately 10-fold, leading to a decrease in protein kinase G (PKG) stimulation and ultimately affecting vascular tone and growth in erectile dysfunction [71]. The inhibition of PDE5 leads to elevated cGMP and NO levels. 

The 16-week, double-blind, phase III clinical trial that led to the approval of tadalafil for pulmonary arterial hypertension treatment consisted of 405 patients diagnosed with PAH. The patients were given a prescribed dosage of tadalafil or a placebo, and their performance to walk for six minutes was evaluated based on the distance they covered [70]. The findings of the study identified that an oral dosage of 40 mg per day of tadalafil could improve exercise capability by an average of 33 m in patients with pulmonary arterial hypertension versus the placebo patients. Dosage was weight-dependent, though all patients weighed an average of 75 ± 22 kg in this study. Of the 357 patients that participated in a long-term study with tadalafil, 295 of them continued treatment. While this study, along with other clinical trials, showed vast improvements in patients with pulmonary arterial hypertension, the molecular mechanism by which tadalafil acts as a therapeutic has yet to be elucidated.

## 4. Modulating Autophagy in Neurodegeneration

Neurodegenerative disorders are devastating, irreversible conditions that are characterized by the loss of neurons in the brain. In many cases, the onset of neurodegeneration associated with diseases including Parkinson’s and Alzheimer’s disease, is sporadic with no known genetic or environmental cause [72,73]. Given the absence of a causative agent, there are no disease-modifying treatments available, and existing drugs serve only to treat symptoms in an attempt to slow the effects of disease progression [74]. There exists an important need for treatment options that prevent, slow, or stop the progression of neurodegenerative disorders. 

Neurodegenerative disorders can affect different areas of the nervous system and cause patients to exhibit different symptoms depending on the disease; however, many share important characteristics outside the gradual loss of neurons over time. For example, Alzheimer’s disease (AD), Parkinson’s disease (PD), Amyotrophic Lateral Sclerosis (ALS) and Huntington’s disease all exhibit protein aggregate pathology in the form of neurofibrillary tangles, Lewy bodies, inclusion bodies, and mutant Huntingtin aggregates, respectively [75]. It is believed that these aggregates are toxic and play a role in neuronal death. Neurodegenerative disorders are also commonly associated with aberrant activation of the innate and adaptive immune systems in the central nervous system. Currently, significant research efforts are focused on targeting the mechanism by which these aggregates are removed by cell, namely autophagy. Alternatively, efforts also include studying the role inflammation plays in these disorders [71].

Autophagy is a cellular process responsible for maintaining protein homeostasis in the cell. While there is more than one mechanism of autophagy, macro-autophagy is the most studied and best understood type. For this reason, we will focus on macro-autophagy (hereafter referred to as autophagy). In autophagy, cytoplasmic materials such as proteins, metabolites, and organelles are engulfed by membrane-bound vesicles known as autophagosomes. These autophagosomes are then trafficked to a lysosome, which then fuses with the autophagosome and is then degraded and released [75].

**Figure 4:**
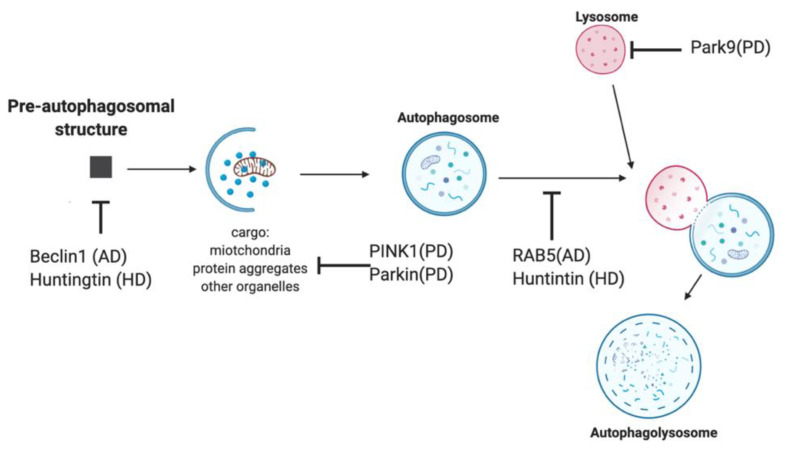
Autophagy mechanism and associated gene dysfunctions. Autophagy is a cellular mechanism by which metabolites, organelles, proteins and protein aggregates are enveloped by a vesicular membrane to form an autophagosome. This autophagosome is trafficked to a lysosome where fusion occurs, and lysosomal degradative enzymes break down the cargo. Dysfunction in several genes associated with neurodegenerative diseases has been implicated and are known to disrupt autophagy. The schematic image (Figure 4) was created using BioRender.com.

A hallmark of neurodegenerative diseases is the accumulation of protein aggregates. Autophagy is a known mechanism to clear large aggregates of protein and provides potential cellular targets for a disease-modifying treatment. Further, dysfunction in autophagy and its regulation have been implicated in many common neurodegenerative diseases [72]. In PD, mutations in PTEN-Induced Kinase 1 (PINK1) and Parkin, an E3 ubiquitin ligase involved in mitochondrial turnover, are known to disrupt the autophagic degradation of mitochondria leading to stress-induced mitochondrial dysfunction and cell death [71]. Additionally, mutations in Park9 disrupt acidification in the lysosome [76]. In AD, reductions in expression of Beclin 1, a protein involved in the induction of autophagy, and Rab5, an endosomal and lysosomal regulator, have been implicated in the progression of the disease [76]. Finally, in Huntington’s disease, huntingtin protein is involved in various aspects of autophagy, such as cargo recognition, endosomal and lysosomal regulation, and vesicular trafficking, and its canonical polyglutamine tract mutation has been shown to interfere with these regular functions (of autophagy) (Figure 4) [76]. Felodipine and lonafarnib are two examples of drug repurposing candidates that have undergone study for their activity to target autophagy.

### 4.1. Felodipine

Felodipine is an antihypertensive drug originally approved by the FDA in 1988 [77]. It prevents hypertension by inhibiting L-type calcium channels and preventing calcium-dependent smooth muscle contractions, namely those responsible for constriction of the blood vessels [77]. Currently, felodipine is being investigated as an inducer of autophagy in Huntington’s disease and Parkinson’s disease models. 

Calcium influx inhibition induces autophagy by reducing the activity of calcium-dependent cysteine proteases, which are also known as calpains. Upon activation, calpains cleave G-proteins, which results in the activation of adenylyl cyclase. Activated adenylyl cyclase increases the amount of cyclic AMP (cAMP) in the cell and activates phospholipase C. Phospholipase C is responsible for the generation of phosphatidylinositol 3-phosphate (PIP3), which is a direct inhibitor of autophagy and allows more calcium to enter the cell, creating a positive feedback loop. By blocking cytoplasmic calcium, there is a net reduction in PIP3 and an induction of autophagy [78].

Felodipine activity to induce autophagy by blocking L-type calcium channels was identified as favorable in a screen of drugs assessed in primary mice neurons [79]. Out of nine calcium channel blockers, felodipine showed the greatest increase in autophagosomes and autophagolysosomes. The efficacy of felodipine has been further assessed in zebrafish models of tauopathy, a class of neurodegenerative disease involving the aggregation of Tau protein in the human brain, mouse models of Huntington’s disease, and mouse models of PD [79]. In mice, felodipine has the ability to ameliorate signs of Huntington’s disease over a treatment period of 12 weeks and resulted in improved clearance of alpha-synuclein, the main component of Lewy bodies. Twenty-two B6HD mice, a Huntington’s disease model, were treated with felodipine starting in their sixth week from birth, and were tested for grip strength, wire maneuvering and tremors, every two weeks from age 7 to 19 weeks. Felodipine-treated mice were reported to have better grip strength from week 11–19 and a reduction in tremors from weeks 17–19, when compared to vehicle-treated control mice [79]. Likewise, A53T alpha-synuclein mice, a common PD mouse model, were shown to have improved grip strength, increased neuron numbers in the substantia nigra (the area of the brain affected by PD), and a reduction in insoluble alpha-synuclein, a protein that comprises Lewy bodies. In zebrafish, a rho: tau model was used to emulate cell death associated with the aggregation of Tau protein commonly associated with AD. In this model, wild-type human tau protein is expressed in rod photoreceptors and causes cell death. Felodipine successfully rescued rod cell death, but failed to do so in zebrafish lacking atg7, a vital autophagy gene, suggesting the effect of felodipine on these cells is dependent on autophagy [79].

While felodipine has shown promise as a potential new treatment for neurodegeneration, cautious skepticism is advised due to how recent the studies have been conducted and the scope of studies has been limited to preclinical models of disease. Human clinical trials and efficacious dosage regimens remain to be conducted. Due to differences between human and mouse physiology, felodipine is metabolized much quicker in mice. As a result, the mice in this study were exposed to transient exposures of felodipine that are two orders of magnitude higher than the dosage indicated for use as an antihypertensive drug in humans. It is possible that the required dosage is so high that deleterious side-effects may preclude human treatment. Finally, the models used in this work were only successful in showing that felodipine can clear protein aggregates; the studies did not include the full disease course or its progression. 

### 4.2. Lonafarnib

Lonafarnib is a promising drug that has passed phase two clinical trials for the treatment of progeria, a genetic disorder causing accelerated aging in children, and phase three clinical trials for the treatment of hepatitis delta virus infections [80]. Lonafarnib is a farnesyltransferase inhibitor that prevents prenylation, the addition of a lipid group to the cysteine residues of proteins. In hepatitis delta infections, prenylation is required for the assembly and packaging of new viral particles [79]. By inhibiting prenylation, fewer viral particles are created. In progeria, a genetic mutation causes Lamin A to be truncated and form progerin. Lamin A is an important part of the nuclear membrane structure and may be required for the repair of double-stranded DNA breaks [81]. When progerin is formed instead, it is prenylated and cannot incorporate into the nuclear membrane. By inhibiting farnesyltransferase and prenylation, the instability of the nuclear membrane can be rescued [81].

Interestingly, lonafarnib shows promise as an inducer of autophagy and might be a possible therapeutic to treat tauopathy. In the cell, autophagy is regulated by the mechanistic target of rapamycin (mTOR) kinase, also commonly referred to as FK506-binding protein 12-rapamycin-associated protein 1 (FRAP1). When mTOR is active, autophagy is inhibited. mTOR itself is regulated by the G-protein, Rheb, which is functional when localized to the cell membrane via a fatty acid chain tether. Farnesyltransferase inhibitors, such as lonafarnib disrupt the prenylation of Rheb and prevent its localization to the inner cell membrane. Without Rheb in the proper position, mTOR cannot be activated, resulting in an upregulation of autophagy [82].

Lonafarnib has been shown to induce autophagy in primary mice fibroblast cells as well as improving symptoms in mice models of dementia. In a study by Hernandez et al., transgenic NIH3T3 mouse fibroblast cells were used to show efficacy in inducing autophagy [83]. NIH3T3 cells were used to perform a fluorescent assay that can determine the rate of autophagy [82]. NIH3T3 cells treated with increasing doses of lonafarnib exhibited dose-dependent increases in autophagolysosomes suggesting a lonafarnib-mediated increase in autophagy. Furthermore, Tg4510 mice, used as a dementia model displaying behavioral impairments associated with dementia, were treated with lonafarnib starting at 10 weeks of age with oral administration of lonafarnig at 80 mg/kg per day. At 20 weeks, mice were assessed for nest-building behaviors. Treated mice displayed an improvement in nest shredding, a trait associated with these mice. Lonafarnib was also able to ameliorate the circling behavior attributed to this model, but not reverse it after already manifesting. Due to these findings, it was concluded that lonafarnib cannot reverse existing tau pathology, but can prevent it from worsening [83].

While lonafarnib is indeed a promising drug, it is important to consider that this drug is still in relatively early stages of the drug discovery pipeline. It has not yet been approved by the FDA and is not clinically available. It is also important to note that the successful use of this drug as a treatment for dementia would also be hindered by the fact that lonafarnib cannot reverse the damage done by tauopathy. Because of this, lonafarnib may be essential in patients with early diagnoses. Studies on the inhibition of prenylation of proteins should be performed to determine if lonafarnib may have any off-target effects, as there are many proteins that need to be prenylated to function correctly. It is likely that in the case of progeria, the life-saving effect seen by long-term use of this drug outweighs any potentially harmful side effects, but the same cannot be inferred for dementia. 

## 5. Inflammation and NRF2 as a Target in Neurodegeneration

**Figure 5:**
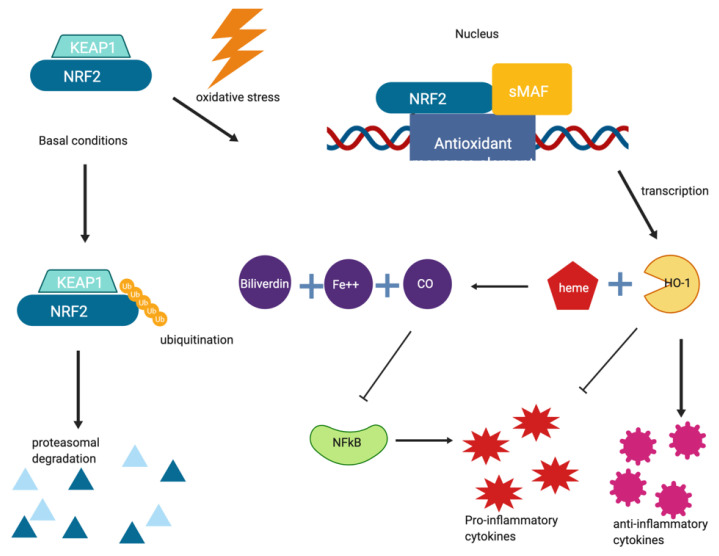
NRF2 signaling and inflammation. Under basal conditions, NRF2 is bound to its repressor, KEAP1 and ultimately degraded by the proteasome following ubiquitination. However, under oxidative stress, free NRF2 translocates to the nucleus and dimerizes with small MAF family proteins. This complex binds to and promotes the expression of genes with an antioxidant response element, such as HO-1. HO-1 directly inhibits pro-inflammatory cytokines while upregulating anti-inflammatory cytokines as well as catalyzing the breakdown of heme into carbon monoxide, free iron, and biliverdin. Carbon monoxide is an inhibitor of the NFκB pathway, resulting in an overall decrease of pro-inflammatory cytokines. The image in Figure 5 was created using BioRender.com.

Chronic inflammation in the brain is associated with many neurodegenerative disorders including multiple sclerosis, Parkinson’s disease, macular degeneration, cutaneous T-cell lymphoma, obstructive sleep apnea and adult brain glioblastoma and rheumatoid arthritis [71]. It has been hypothesized that the innate immune response and chronic inflammation of the brain mediated by microglia and astrocytes have a role in the onset of neurodegeneration, and there is interest in studying therapeutics to reduce neuroinflammation. As an example, nuclear factor (erythroid-derived 2)-like2 (NRF2) is a transcription factor that regulates the expression of detoxifying enzymes and antioxidant genes including NAD(P)H quinone oxidoreductase 1, heme oxygenase-1(HO-1), glutathione S-transferase (GST) genes, and UDP-glucuronosyltransferase (UGT) genes. NRF2 is inhibited by KEAP1 under normal conditions, but NRF2 is activated under stressful conditions. Under normal conditions, KEAP1 binds to NRF2 and mediates its degradation via the ubiquitin-proteasome system. However, under conditions of oxidative stress, KEAP1 releases NRF2 allowing it to upregulate any number of antioxidant genes. One such gene is HO-1. HO-1 catalyzes the degradation of heme into carbon monoxide (CO), free iron, and biliverdin as well as directly upregulating anti-inflammatory cytokines and inhibiting pro-inflammatory cytokines. In addition to these points, CO inhibits NFκB, a signaling protein responsible for promoting pro-inflammatory cytokines [84]. Activation of the NRF2 signaling pathway, which is shown in Figure 5, has a net anti-inflammatory effect. Dimethyl fumarate and exemestane are two drugs that could be repurposed for treatment of inflammation due to aberrant NRF2 signaling pathways. 

### 5.1. Dimethyl Fumarate

**Figure 6:**
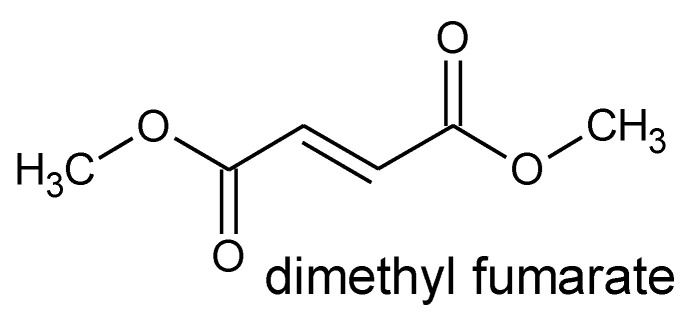
Chemical structure of dimethyl fumarate.

Fumaric acid derivatives were first utilized in the 1950s for the treatment of psoriasis. The mechanism of action for fumaric acid and its derivatives was thought to be that a combination of oral and topical administration of these compounds restored an imbalance in the citric acid cycle, as fumaric acid is an intermediate product in the cycle, and a fumarate deficiency was proposed as a problem in patients with psoriasis [85]. However, early clinical trials could not reproduce these results and fumarates were discontinued for over ten years. In the early 1990s, the first clinical trial for dimethyl fumarate (DMF) was conducted for the treatment of psoriasis and the results proved favorable in regards to severe plaque psoriasis [85]. The topical, exogenous administration of dimethyl fumarate (Figure 6) evolved over time into an oral formulation in the late 1990s. In 1994, the EU approved DMF in combination with monoethyl fumarate salts for usage in Germany as an oral treatment for moderate to severe psoriasis [86]. 

DMF was approved by the FDA and EMA in 2014 as a medication to treat relapsing forms of multiple sclerosis (MS) at 240 mg 2–3 times per day [87,88]. Multiple sclerosis is an autoimmune disease that is characterized by the degradation of the myelin sheath surrounding nerves, resulting in pain and motor function impairment. DMF is metabolized to monomethyl fumarate in the body, which acts as an agonist of the nAChR, resulting in activation of the NRF2 pathway leading to a reduction in the inflammation that exacerbates demyelination. More recently, DMF has been found to activate this same anti-inflammatory pathway in PD patients, providing a promising treatment option for neurodegeneration that appears to work in mouse models of α-synucleinopathy at 100 mg/kg [89]. In PD, DMF has demonstrated a neuroprotective effect by ameliorating mitochondrial dysfunction and upregulating mitochondrial biogenesis by blocking neurotoxicity in wild-type, but not NRF2 knockout mice [90].

On par with other immunomodulation treatments, DMF does not have a singular mechanism of action or one biochemical pathway that is targeted with administration. Rather, it is believed that DMF has multiple targets and exerts a spectrum of biological effects that lead to the therapeutic experienced by MS patients treated with DMF [86]. As shown in in vitro studies, DMF partially exerts anti-inflammatory action with the ability to increase the production of interleukin-4 (IL-4) and IL-5 when added to cultures of stimulated peripheral mononuclear blood cells [91]. These cytokines are conducive to producing and promoting a Th2 immune response. The perturbation away from the Th1 response was also replicated in dendritic cells. The polarization of the immune response away from Th1 and toward the Th2 profile is a probable mechanism for DMF function as an immunomodulator. There is also evidence that DMF stimulates the native anti-oxidative stress machinery in cells. The antioxidative response is activated by NRF2, the primary transcription factor for genes associated with the anti-oxidative response. DMF has been shown to enhance the nuclear translocation of NRF2, and thus activating transcription of associated genes. The effect DMF has on the NRF2 pathway is likely a profound contributor to the mechanism of action [87].

In a study by Lastres-Becker et al., rAAV6-ɑ-synuclein NRF2^+/+^ and NRF2^−/−^ mice were treated with DMF for one and three weeks followed by additional treatments every other day for eight weeks [89]. One day before sacrifice, the mice were assessed for motor asymmetry via the elevated body swing test. The NRF2^−/−^ mice displayed significantly increased contralateral body torsion compared to the NRF2^+/+^ mice [92]. Additionally, DMF was assessed for its effect on signs of inflammation at the tissue level. One sign of inflammation in Parkinson’s disease is the increase of microglia and astrocytes in the basal ganglia known as microgliosis and astrocytosis, respectively. In order to test the effect of DMF on these inflammatory markers, NRF2^+/+^ mice treated with DMF displayed significant decreases in astrocytosis and microgliosis compared to the NRF2^−/−^ mice. In the brain, microglia can express one of two phenotypes, a pro-inflammatory, IL-1β and inducible nitric oxide synthase (iNOS)-producing phenotype and anti-inflammatory, IL-4 and sphingosine kinase 2-producing phenotype. DMF was shown to mediate the conversion of the pro-inflammatory phenotype to the anti-inflammatory phenotype in mouse BV2 microglial cells. Cells treated with DMF displayed increased levels of IL-4 mRNA in a time-dependent manner [89]. DMF was also shown to have a neuroprotective effect on BV2 cells in studies where the cells were pretreated with DMF and then exposed to ɑ-synuclein. Untreated cells had an induction of IL-1β and iNOS whereas pretreated cells displayed lower pro-inflammatory marker induction. Based on these findings, NRF2 appears to be a potentially useful target for Parkinson’s disease treatment, and DMF may bring us one step closer to a disease-modifying drug [92].

A survey of clinical trial data yielded that DMF has been or is being investigated for efficacy in treating the following disease states: primary progressive multiple sclerosis, relapsing-remitting multiple sclerosis, age-related macular degeneration, cutaneous T-cell lymphoma, obstructive sleep apnea, adult brain glioblastoma and rheumatoid arthritis. While there is a diversity of pathologies being investigated with the administration of DMF, inflammation arises as a shared theme in these clinical trials. Immunomodulation, downregulation of proinflammatory cytokines, the effect of DMF on T-cell regulation, and DMF exerts in vitro could be of utility in treating a myriad of diseases [93].

### 5.2. Exemestane

Exemestane was approved by the FDA and EMA in 1999 and is an aromatase inhibitor for use on estrogen-dependent breast cancers, especially in post-menopausal women at a dosage of 25 mg per day [94,95]. Exemestane treats breast cancer in post-menopausal women by preventing the synthesis of estrogen via the inhibition of the aromatization step of androgens into estradiol, a precursor of estrogen. Without estrogen, these estrogen-dependent cancers’ growth and spread is inhibited. Exemestane was identified as a potential treatment for Parkinson’s disease through a screen aiming to find compounds that activate HO-1 [96]. Exemestane was found to be one of the most effective compounds in the screen. The proposed mechanism for treating Parkinson’s disease is different than that of treating breast cancer, where exemestane inhibits the formation of estrogen from androgen during the rate-limiting step of aromatase conversion [93,97]. In this sense, exemestane acts as an upregulator of NRF2 expression, a transcription factor responsible for producing antioxidant enzymes in order to circumvent the degeneration of dopaminergic neurons due to high amounts of oxidative stress from ROS such as NO [94,98].

In a study by Son et al. (2017), exemestane was validated in BV2 microglial cells and in MPTP mouse models of Parkinson’s disease. BV2 cells were tested for NRF2 protein levels, HO-1 and NQO1 mRNA levels, and iNOS and IL-1β levels following exemestane treatment [94]. NRF2 expression was elevated 4.4-fold, suggesting exemestane-mediated activation. Likewise, the expression of NRF2 downstream genes increased with exemestane treatment, while inflammatory marker levels decreased. Taken together, these results signify a possible reduction of inflammation by exemestane. In the same study, exemestane was also evaluated in a mouse model of Parkinson’s disease. Mice were treated with MPTP, a neurotoxin that kills dopaminergic neurons and attempts to simulate the death of neurons in Parkinson’s disease. The mice were then co-administered with exemestane orally (1, 10, or 20 mg/kg), three times a day, every day for seven days. Following treatment, the mice were sacrificed. Immunostaining was performed for tyrosine hydroxylase, a selective marker expressed by dopaminergic neurons. Mice co-treated with exemestane exhibited a 42% reduction in the loss of dopaminergic neurons caused by MPTP [94].

DMF and exemestane both show enticing results as potential new treatments for Parkinson’s disease; however, the exact molecular target of either drug remains unknown. The Parkinson’s disease model studies performed with DMF and exemestane are still preclinical, limiting any interpretation of results regarding potential efficacy in humans. 

One concern that may be raised for exemestane experiments has to do with the MPTP mouse model. The addition of a toxin does not necessarily recapitulate Parkinson’s disease progression because the neurons die all at once instead of gradually over time as they would in human disease progression. Additionally, the results from the Son et al. study seem to contradict previous reports that estradiol has a neuroprotective effect in PD [94]. However, estradiol and its structural analog, exemestane, exhibit their neuroprotective qualities through JNK and NRF2 pathways, respectively. This would necessitate further studies on the effects of estradiol and its inhibition in the context of PD. Another consideration that must be made is that the experiments conducted on mice for both drugs were done over a relatively short period of time. The DMF treatments lasted eight weeks and the exemestane tests were done for seven days. It will be necessary to discover how long-term treatments affect the progression of Parkinson’s disease in more relevant model systems up to and including human trials.

## 6. Conclusions

The benefit of repurposing drugs that were previously approved by the FDA removes many of the time and financial barriers for bringing a drug to market. FDA approval is a good indication of a drug’s effectiveness and safety in clinical trials, which is indicative of a good outcome for the use of a drug for a different purpose. Because a drug can take anywhere from 8–12 years from its initial discovery to the market, drug repurposing can reduce this time significantly. The repurposing of drugs to treat cardiovascular and neurodegenerative diseases is an emerging and promising field of study. Treatments for inflammatory diseases including vascular endothelial dysfunction, pulmonary arterial hypertension, multiple sclerosis, Parkinson’s and Alzheimer’s, using repurposed drugs like colchicine, methotrexate, tocilizumab, felodipine, lonafarnib, dimethyl fumarate and exemestane may prove to be an effective strategy to address the treatment gap for untreatable ailments.

## Figures and Tables

**Table 1:** Examples of some FDA-approved repurposed drugs.

Drug	Original Purpose	Repurposed Use	Reference
Amantadine	Parkinson’s disease	Influenza A	[4]
Acetylsalicylic acid	Inflammation, pain relief	Antiplatelet	[5]
Cyclosporine	Rheumatoid arthritis	Transplant rejection, psoriasis, chronic dry eye	[6]
Minoxidil	Hypertension	Alopecia	[7]
Imatinib	Chronic myelogous leukemia	Gastrointestinal stromal tumor	[8]

**Table 2:** Examples of drug or drug targets that have been identified using computational methods.

Drug or Target	Target Pathology	Research Method	Reference
kinase inhibitors	Alzheimer’s disease	machine learning	[32]
dopaminergic agonists	Parkinson’s disease	machine learning	[24]
beta-secretase 1 inhibitors	Alzheimer’s diseases	data mining	[33]
GIST-T1 cells	Gastrointestinal stromal tumor	High-throughput synergy screening	[34]
